# A Virtual Community of Practice for General Practice Training: A Preimplementation Survey

**DOI:** 10.2196/mededu.5318

**Published:** 2016-08-18

**Authors:** Stephen Barnett, Sandra C Jones, Sue Bennett, Don Iverson, Laura Robinson

**Affiliations:** ^1^ General Practice Academic Unit University of Wollongong Wollongong Australia; ^2^ Centre for Health and Social Research Australian Catholic University Melbourne Australia; ^3^ School of Education University of Wollongong Wollongong Australia; ^4^ Faculty of Health, Arts and Design Swinburne University Melbourne Australia; ^5^ Centre for Health Initiatives University of Wollongong Wollongong Australia

**Keywords:** medical informatics, e-learning, virtual communities of practice

## Abstract

**Background:**

Professional isolation is an important factor in low rural health workforce retention.

**Objective:**

The aim of this study was to gain insights to inform the development of an implementation plan for a virtual community of practice (VCoP) for general practice (GP) training in regional Australia. The study also aimed to assess the applicability of the findings of an existing framework in developing this plan. This included ascertaining the main drivers of usage, or usefulness, of the VCoP for users and establishing the different priorities between user groups.

**Methods:**

A survey study, based on the seven-step health VCoP framework, was conducted with general practice supervisors and registrars—133 usable responses; 40% estimated response rate. Data was analyzed using the t test and the chi-square test for comparisons between groups. Factor analysis and generalized linear regression modeling were used to ascertain factors which may independently predict intention to use the VCoP.

**Results:**

In establishing a VCoP, facilitation was seen as important. Regarding stakeholders, the GP training provider was an important sponsor. Factor analysis showed a single goal of usefulness. Registrars had a higher intention to use the VCoP (*P*<.001) and to perceive it as useful (*P*<.001) than supervisors. Usefulness independently predicted intention to actively use the VCoP (*P*<.001). Regarding engagement of a broad church of users, registrars were more likely than supervisors to want allied health professional and specialist involvement (*P*<.001). A supportive environment was deemed important, but most important was the quality of the content. Participants wanted regular feedback about site activity. Regarding technology and community, training can be online, but trust is better built face-to-face. Supervisors were significantly more likely than registrars to perceive that registrars needed help with knowledge (*P*=.01) and implementation of knowledge (*P*<.001).

**Conclusions:**

Important factors for a GP training VCoP include the following: facilitation covering administration and expertise, the perceived usefulness of the community, focusing usefulness around knowledge sharing, and overcoming professional isolation with high-quality content. Knowledge needs of different users should be acknowledged and help can be provided online, but trust is better built face-to-face. In conclusion, the findings of the health framework for VCoPs are relevant when developing an implementation plan for a VCoP for GP training. The main driver of success for a GP training VCoP is the perception of its usefulness by participants. Overcoming professional isolation for GP registrars using a VCoP has implications for training and retention of health workers in rural areas.

## Introduction

Professional isolation is an important factor in low rural health workforce retention [1]. Isolation can lead to decreased knowledge sharing [2] and can affect the career choices of doctors, including intending to work reduced hours and moving away from rural areas [3-5]. Training for doctors in general practice in Australia can be particularly isolating [3,4]; trainees, or registrars, can be spread across large geographic areas, moving between different practices in urban and regional placements, and are usually alone in their consulting room with a patient. These factors of geography and structure are barriers to knowledge sharing, impeding the natural communities of practice that form in medical training.

Communities of practice (CoPs) are “groups of people who share a concern or a passion for something they do and learn how to do it better as they interact regularly” [6]. CoPs reflect the master and apprentice knowledge sharing that occurs between senior doctors and those in training. In knowledge management terms, there are two types of knowledge being shared in this type of master and apprentice learning. Firstly, explicit knowledge sharing occurs around a topic; for example, the details of which drugs are appropriate for a clinical condition [7]. This can be referred to as the *know-what*. Secondly, and most importantly, CoPs help participants share tacit knowledge [7]. This is the *know-how* of putting that knowledge into practice; for example, how to ensure a clinical condition is identified from a primary care database, that the patient is recalled, that patients are encouraged to take medications, and how to anticipate and treat a range of side effects. Through this knowledge transfer, CoPs can lead to significant quality improvement in patient care, such as the establishment of a nationally lauded stroke service in the United Kingdom [8] or the delivery of care to hepatitis C patients in rural areas to the same standard as an academic medical center [9].

More recently, online technology has been enabling medical information sharing on an unprecedented scale [10-12], with doctors around the world joining and using a wide range of online medical communities [13]. As a result, virtual communities of practice (VCoPs) have developed in a number of industries, including health care, in which online technologies are used to overcome barriers of distance and work structure [14-16]. For example, in Canada, emergency department staff share knowledge between rural and urban centers [14], while in primary health care in Spain, the HOBE network has engaged over 1500 primary care professionals in a VCoP for health care innovation, leading to the development and implementation of a number of important service improvement strategies [17].

In this context, two studies have shown that there is the interest, ability, and Internet access among general practice (GP) registrars and supervisors to establish a VCoP for GP training in a regional area of New South Wales, Australia [18,19]. As part of these studies, a health framework for VCoP implementation was developed, based on a review of the business and health care literature [16,20].

The aim of this study was to gain insights to inform the development of an implementation plan for the Virtual Community of Practice (VCoP) for General Practice Training in regional Australia. The VCoP platform was based on the NING social networking software [21], which can be customized to offer a variety of features for private social networks. Users were asked about features such as forums, live chat, shared document repositories, and videoconferencing (see Multimedia Appendix 1). The study also aimed to assess the applicability of the findings of an existing framework in developing this plan. This included ascertaining the main drivers of usage, or *usefulness*, of the VCoP for users and establishing the different priorities between user groups.

## Methods

Ethics approval was obtained from the University of Wollongong’s Human Research Ethics Committee.

### Participants

The sampling frame comprised all general practice registrars, supervisors, and educators in Coast City Country General Practice Training (CCCGPT). CCCGPT provides general practice training in a 160,000 square kilometer region of Australia, covering urban, regional, and small rural centers in the Australian Capital Territory and New South Wales. After 2 hospital years, GP registrars progress through a minimum of three general practice terms of 6 months.

In October 2011, an email was sent to the GP training provider database by the training provider administration, inviting recipients to fill in an online survey. The GP training provider database keeps an accurate record of registrars and their email addresses, listed by date. The registrar sampling frame was 143. The supervisor database is less accurate as supervisors’ details are not updated each term, while registrars’ details are. Supervisor emails are not always updated when they change and there is no date range to retrospectively check when they were active as supervisors or having a break from training. Given these limitations, a manual review by the training provider administration of the list of supervisors within the training program, cross-checked against the training program database, gave a supervisor sampling frame of 175, giving a total registrar and supervisor sampling frame of 318. In the invitation email, there was a link to SurveyMonkey, a Web-based survey program (SurveyMonkey, LLC, Palo Alto, CA, USA), with a survey and participant information sheet. A total of 183 out of 318 people responded, yielding a 57.5% response rate; 50 cases were removed for not providing consent or demographics (n=12) or for not completing the majority of the survey (n=38). Some of these noncompletions were due to emails going to practice management staff rather than doctors. The total usable response rate was 41.8% (133/318): registrar 46.9% (67/143) and supervisor 37.7% (66/175).

### Questionnaire

The questionnaire was based on previous studies demonstrating GP registrar and supervisor interest in a VCoP, and a framework that guides the implementation of health VCoPs [18-20].

The seven steps of the health VCoP framework are as follows: (1) organizing facilitation; (2) engaging stakeholders; (3) establishing clear goals; (4) involving a broad church of participants; (5) creating a supportive environment; (6) including measurement, benchmarking, and feedback in the design; and (7) technology and community factors, such as users self-selecting and having a mixture of face-to-face and online engagements. There were 28 questions in the final survey. Questions included categorical and 5-point Likert scale response items. The questions collected information on each of the seven steps, to investigate whether the steps were applicable to a VCoP for GP training. This included questions in which respondents rated the importance of a step, along with questions seeking further detail on that step to help guide the VCoP implementation. In addition, questions were asked to assess the knowledge needs of registrars when implementing guidelines, so that information on the appropriate content for the site could be obtained. Items about the features of the site were included to determine which tools would be most useful. The survey is included as Multimedia Appendix 1, but it is worth noting that, due to the logic within the online survey, the printed version can appear to have repetitions. In the online survey, participants only received each appropriate question once.

The instrument was piloted with 2 GP registrars, 2 supervisors, and 4 researchers. Discussion among this group led to some minor alterations to clarify wording. Results are presented under the seven headings of the health VCoP framework.

### Statistical Analysis

Data were analyzed using SPSS version 19 (IBM Corp, Armonk, NY, USA). For comparison between groups, respondents were categorized as either registrar or supervisor; *t* test and chi-square analyses were performed. The paired-samples *t* test was used to compare responses within a group. The independent sample *t* test was used to compare categorical and scale data. All statistical comparisons were two-tailed and statistical significance was set at *P*<.05.

Principal axis factor analysis using varimax rotation was used to determine which Likert scale items grouped naturally in questions with multiple Likert scale items; for example, the question on the practical outcomes, or usefulness, that an online network would have for that user. If eigenvalues were >1.0, factors were included. To test for the agreement between the Likert scale items, such as the five factors perceived as *useful* outcomes for a VCoP, and separately for the two items of *intention to actively use the VCoP*, the Cronbach alpha test for reliability was calculated.

General linear regression modeling was used to test the multivariate associations of independent variables such as age, training stage, and usefulness, and the dependent variable of intention to actively use an online network for GP training.

## Results

### Overview

There were 133 medical practitioners in the final sample. Of these, 51.9% (69/133) were male, 57.1% (76/133) were from a rural setting, and 50.4% (67/133) were registrars. Registrars were younger (mean 36.70 years, SD 6.85) than supervisors (mean 52.62 years, SD 7.90; *t* test *P*<.001) and more likely to be female (63% [42/67] female registrars compared with 33% [22/66] female supervisors; chi-square *P*=.001).

### Factor Analysis

To determine which questions in the survey naturally clustered together, principal axis factor analysis using varimax rotation factor analysis was performed on two groups of questions. Participants were asked these questions to verify applicability of *Step 3: Goals and Objectives*, as seen below, and the results will be fully discussed in that step. The factor analysis is described below. Cronbach alpha was >.80, above the recommended threshold of .70 in both cases.

The first question contained five items. Participants were asked what practical outcome, or *usefulness*, such a network would deliver. The five items included helping registrars pass exams, participants feeling more confident in medical skills, learning from colleagues about putting guidelines into practice, feeling more supported in general practice, and developing a broader network of colleagues. These were analyzed using factor analysis and found to be a single factor (Cronbach alpha=.90, eigenvalue=4.01). The single factor covered a broad range of useful outcomes of a network, including support, broad network, improved confidence, and learning skills, and so the factor was labeled *useful for training*, and afterward referred to as *usefulness*.

Secondly, participants were asked about their intention to use an online network for training by ranking their likelihood of participating through reading, sharing knowledge by answering questions, and uploading new topics. The rating scale ranged from 1 (not likely) through to 5 (highly likely). *Only reading* was passive participation. Sharing knowledge by posting new topics and sharing knowledge by answering questions were both methods of active participation. Factor analysis of these two active participation questions revealed a single factor, *likelihood to use actively* (eg, posting and starting topics). For analysis purposes, the question on passive participation is referred to as *likelihood to use passively* (*only reading*).

### Health Virtual Community of Practice Framework Step 1: Facilitation

Facilitators promote engagement and maintain community standards. [20]

Respondents (N=132) rated the need for formal facilitation between *important* (62/132, 47.0%) and *very important* (32/132, 24.2%). Mean scores were calculated for each group. Analysis using a *t* test showed there were no differences between the importance of formal facilitation for registrars and supervisors (*t*_130_=-0.79, *P*=.43). The most popular choice for community facilitator/leader was a topic expert (53/133, 39.8%), with registrars rating a topic expert as highest and supervisors rating a topic expert second behind the training provider administration. Chi-square testing showed that registrars were significantly more likely to want a topic expert than were supervisors (*P*=.03), with no statistical significance between other results (see Table 1).

**Table 1:** Preferred leader/facilitator for the online training network (multiple responses allowed).

Facilitator	Registrar (n=67), n (%)	Supervisor (n=66), n (%)	Significance (chi-square test)
Topic expert	33 (49)	20 (30)	Yes: *P*=.03
GP^a^registrar liaison	14 (21)	8 (12)	No
GP supervisor	14 (21)	12 (18)	No
GP training provider administrator	19 (28)	24 (36)	No
Network developer (IT^b^)	5 (7)	5 (8)	No
Network designer (doctor)	18 (27)	17 (26)	No

^a^GP: general practice.

^b^IT: information technology.

There were nine comments in the *other* section. Regarding who would make the best facilitator, two comments showed participants were unsure who would make the best one, four comments were variations of “someone with medical knowledge,” one was “someone savvy with online leadership,” one was “someone with lots of time,” and another suggested that facilitation could be rotated.

### Health Virtual Community of Practice Framework Step 2: Champion and Support

The network needs to have an initial stakeholder champion, with stakeholder support. [14]

Respondents (N=130) rated the need for formal support from the main stakeholder, the GP training provider, between *somewhat important* and *important* (mean 3.73, SD 1.09). The *t* test analysis showed no significant differences between registrars and supervisors (*t*_128_=-0.44, *P*=.66).

The importance of the GP training provider’s support was also reflected in the previous step (see Table 1), in which supervisors rated the GP training provider as the preferred choice of leader/facilitator, whereas the GP training provider was the second preference for registrars. A comment by one respondent supports stakeholder involvement, suggesting that the GP training provider’s medical educator should be the leader/facilitator.

### Health Virtual Community of Practice Framework Step 3: Objectives and Goals

Clear objectives provide members with responsibilities and motivate them to contribute more actively. [20]

Participants were asked about a range of goals for the network, and the key goal, as identified by factor analysis discussed above, was *usefulness*. The perceived usefulness varied between user groups, being significantly higher among registrars (mean 4.11, SD 0.73) than among supervisors (mean 3.44, SD 0.82; *t*_131_=4.98, *P*<.001). A thematic analysis by the first author (SB) of the 25 comments about specific goals showed an even split between concepts of knowledge sharing and improving connectedness/overcoming isolation. Knowledge sharing comments focused on sharing information about medicine, employment opportunities, or just being able to exchange information. Examples included “staying up to date with medical knowledge,” “easy to communicate and exchange information,” and “knowing about local services available.” The isolation comments included several participants wanting to “reduce isolation,” “keep in contact with other registrars,” and “debrief,” and noted that such a network would be “particularly important for rural and time-poor colleagues.”

From the health VCoP framework, clear goals are supposed to encourage active participation. Registrars (mean 3.00, SD 1.14) were more likely to state that they would participate actively than did supervisors (mean 2.52, SD 0.87; *t*_131_=4.08, *P*<.001), while there was no difference between supervisors and registrars intending to participate passively (*t*_131_=0.02, *P*=.99).

A multivariate generalized linear regression model was developed using *intention to use actively* as the dependent variable, as active use is the most important driver in establishing an online community. Variables of age, training stage, gender, rurality, and usefulness were included. Perceived usefulness was the only factor significantly predictive of intention to use the network actively (*F*_1_= 29.46, *P*<.001).

### Health Virtual Community of Practice Framework Step 4: A Broad Church

Consider involving different, overlapping but not competing, professional groups, different organisations and external experts. However make sure the church is not too broad... [20]

**Figure 1:**
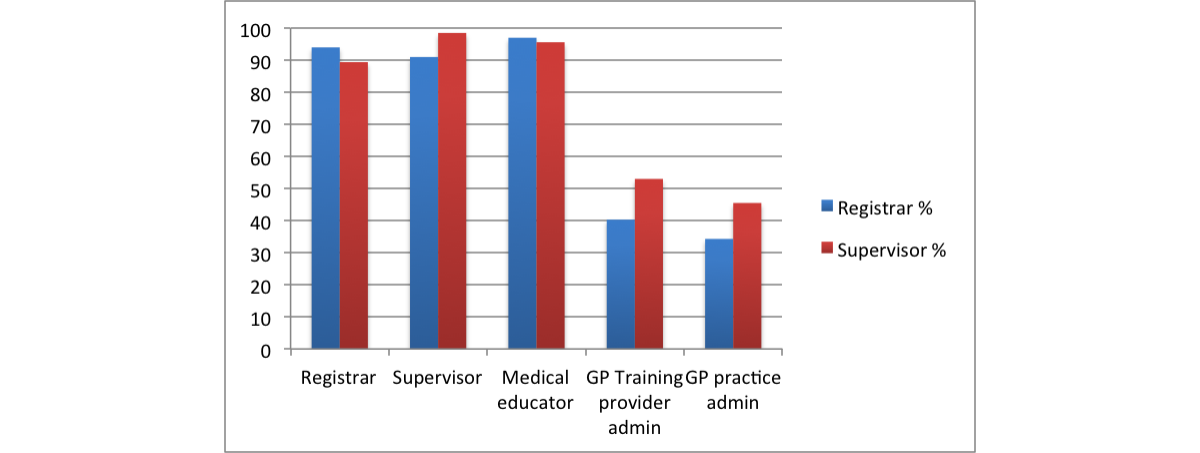
Percentage of respondents supporting participant involvement within training provider. GP: general practice.

Respondents were supportive of a broad church of participants. The inclusion of all medical clinicians within the training provider, including GP registrars, supervisors, and medical educators, was highly supported (see Figure 1), with much less support for the involvement of administrators.

**Figure 2:**
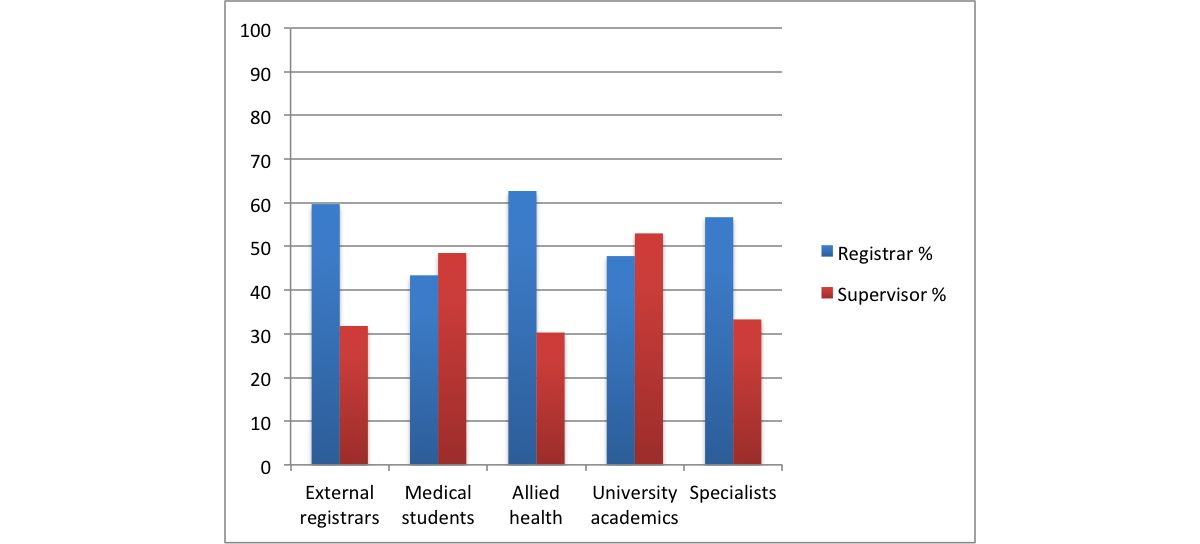
Percentage of respondents supporting participants outside training provider.

There was also much less support for participation from groups outside the training provider, including specialists, students, academics, allied health professionals, and external registrars (see Figure 2). However, registrars (mean 0.63, SD 0.49) were significantly more likely than supervisors (mean 0.30, SD 0.46) to want allied health professionals (*t*_131_=3.93, *P*<.001). Further, registrars (mean 0.57, SD 0.50) were also significantly more likely than supervisors (mean 0.33, SD 0.48) to want specialists in the network (*t*_131_=2.77, *P*=.01).

### Health Virtual Community of Practice Framework Step 5: A Supportive Environment

Health VCoPs should promote a supportive and positive culture that is both safe for members, and encouraging of participation. [14]

Respondents were asked about the aspects that would keep them participating in an online network, including content quality, strength of relationships, financial rewards, continuing education points, and an online points system. Respondents rated the quality of online content as their first preference (mean 4.20, SD 0.63), and their second preference was the strength of the online interaction (mean 3.98, SD 0.73). The preferences in both the registrar and supervisor groups were the same.

### Health Virtual Community of Practice Framework Step 6: Measurement Benchmarking and Feedback

Health VCoPs should consider measurement as a factor in their design, including benchmarking and feedback.[20]

Receiving emails from the community, such as comments, updates, and responses to posts, is termed *feedback* in this context. This feedback can be a useful method of users benchmarking their own knowledge against that of other users, by being directed to updates and responses.

When asked how often respondents would like to be notified that another member had added information, registrars wanted notifications more frequently than supervisors. As shown in Table 2, for registrars, the most common frequency periods for notifications were 1-2 times a week, followed by fortnightly, and then 3-4 times a week. The largest group of supervisors wanted to be notified monthly, followed by 1-2 times a week, and then fortnightly.

Registrars also wanted more frequent notification than supervisors for comments being made on a topic that they had posted. Around half (34/67, 51%) of the registrars wanted to be notified every time a comment was made, compared with only 40% (26/65) of supervisors.

### Health Virtual Community of Practice Framework Step 7: Technology and Community

Online CoPs should ensure ease of use and access, along with asynchronous communication. Other options including chat and meetings can also be considered, along with the need for training. Communities are more likely to share knowledge when there is a mixture of online and face-to-face meetings, members self-select, and both passive and active users are encouraged. [20]

Communities of practice rely on experts and novices sharing knowledge. Respondents were asked how comfortable they were sharing their knowledge. Registrars and supervisors were both comfortable sharing knowledge with colleagues in the training program, although *t* test analysis showed that supervisors (mean 4.32, SD 0.50) were significantly more comfortable than registrars (mean 4.06, SD 0.42; *t*_131_=-3.2, *P*=.002).

VCoP research states that knowledge sharing is best achieved by a mixture of face-to-face and online interaction [16]. As shown in Table 3, results from this study were consistent with VCoP research, as the most common method was a mixture, followed by face-to-face, and then online only. Similar results were found for receiving professional support, with most preferring a mixture of face-to-face and online, followed by face-to-face, or online only.

**Table 2:** Comparisons between registrars and supervisors on notifications from the site.

Question		Registrar, n (%)	Supervisor, n (%)
**How often would you like to be notified that another member had added information? (registrar n=67; supervisor n=66)**			
	Every day	3 (5)	10 (15)
	3-4 times/week	12 (18)	8 (12)
	1-2 times/week	29 (43)	20 (30)
	Fortnightly	17 (25)	16 (24)
	Monthly	5 (8)	21 (32)
**Would you like to be notified every time a comment is made on a topic that you have posted on? (registrar n=67; supervisor n=65)**			
	Yes	34 (51)	26 (40)
	No	12 (18)	24 (37)
	Not sure	21 (31)	15 (23)

**Table 3:** Preference for site-related material, support, and knowledge (N=133).

Question		n (%)
**How would you like to share knowledge?**		
	Purely face-to-face	20 (15.0)
	Purely online	9 (6.8)
	A mixture of online and face-to-face	104 (78.3)
**How would you prefer to receive professional support?**		
	Purely face-to-face	17 (12.8)
	Purely online	6 (4.5)
	A mixture of online and face-to-face	110 (82.7)

Building trust is also important for sharing knowledge. Respondents indicated they were significantly more likely to build trust with other members of their knowledge-sharing community through face-to-face interaction (see Table 4), with no significant difference between registrars and supervisors. In contrast, for simple information transfer such as *help topics*, respondents significantly preferred online delivery to formal face-to-face training.

In terms of the technology used, the most popular feature was shared documents and guidelines, followed by general discussion forums, private subdiscussion groups, email mailing list, videoconferencing, and lastly live chat (see Table 5). The preferences were identical between the groups.

**Table 4:** Preferred methods of building trust and receiving training.

Preferred methods		Mean (SD)^a^
**Preferred method for building trust**		
	Face-to-face (N=132)	4.19 (0.67)
	Online (N=133)	3.69 (0.80)
**Preferred method of training to use the platform (N=132)**		
	Formal face-to-face training	2.92 (1.33)
	Online help (text and images)	3.59 (1.09)

^a^Likert scale ranges from 1 (not important) to 5 (very important).

**Table 5:** Most desirable features for an online training network (N=133).

Preferred features for the technology	Responses, n	Mean (SD)
Shared documents	131	4.01 (0.82)
Discussion forum (all)	132	3.52 (0.97)
Discussions (private)	130	3.22 (1.01)
Email listservs	130	3.10 (1.13)
Videoconferencing	131	2.90 (1.17)
Live chat	131	2.47 (1.19)

When asked for preferences on site usernames, the most popular choice was to use their own name followed by a choice of pseudonym or real name, then using a pseudonym only (see Table 6). Results also show that having a private password-protected site was the clear preference, compared with no password (see Table 6).

**Table 6:** Preference for usernames and passwords for an online training network (N=133).

Preferences for usernames and passwords		n (%)
**Site username preference**		
	Own name	65 (48.9)
	Pseudonym	5 (3.8)
	A choice	63 (47.7)
**Should the site be password protected?**		
	Yes	120 (90.2)

Finally, to further examine the knowledge-sharing needs of registrars and supervisors, participants were asked about the perceived knowledge needs of registrars. The topics covered 14 broad areas of the curriculum for the first 6 months of GP training. Respondents were asked to rate each topic according to how much help GP registrars needed, firstly, in knowing guidelines and, secondly, in implementing guidelines.

Both groups agreed that registrars needed help with their knowledge of topics, but on a combined measure, supervisors felt more strongly that registrars needed help than did the registrars (see Table 7). This pattern was the same with a combined measure for the implementation of knowledge. Supervisors agreed more strongly than registrars that registrars needed assistance. Overall, both groups agreed that the need for support for knowledge acquisition was more important than the need for support regarding the implementation of knowledge, although the absolute difference was small (see Table 8).

**Table 7:** Knowledge of topic areas covered in the first 6 months of general practice training in Australia.

Support needed for registrar learning	Registrars, mean (SD)	Supervisors, mean (SD)	Mean difference	*P* (*t* test)
Need help with knowledge	3.54 (0.80)	4.37 (0.83)	0.83	.01
Need help with implementation	3.59 (0.81)	4.29 (0.50)	0.70	<.001

**Table 8:** Difference between perceived help needed by registrars for knowledge support versus support for implementation of knowledge.

Support needed for registrar learning	Mean (SD)	Mean difference (SD)	*P* (*t* test)
Need help with knowledge	4.02 (0.76)	0.10 (0.44)	<.001
Need help with implementation	3.92 (0.80)		

When looking at the scores of the 14 individual topics, supervisors and registrars rated the importance of topics differently. For example, supervisors gave *knowledge of consultation management* the highest score of importance out of the topics, while registrars gave it the lowest importance score. Administration and compensable injury consultations were in the top five importance scores for both groups.

## Discussion

### Principal Findings

From these results, it is evident that the findings of the health VCoP framework [20] are relevant to the establishment of a VCoP for GP training. However, the results of this study suggest useful additions to some of the steps that will inform the development of an implementation plan for a GP training network using this approach.

The survey results were supportive of a facilitator for the network, in particular a topic expert. The importance of a facilitator is in keeping with previous literature reviews [22,23], and the recent HOBE study in Spain [17]. In the HOBE study, over 5000 primary care providers were invited to participate in a VCoP to encourage innovations in practice. Facilitation was a key factor in the success of the network. The facilitator in the HOBE network was not necessarily a topic expert, yet the desire for a topic expert fits with CoP theory, in which there is a knowledge gradient between experts and novices [6].

However, topic expertise is not the only desirable attribute in facilitators. In *Step 5: A Supportive Environment*, the quality of the relationships with other members and the supportive culture of the network were also seen as important motivators for use. The establishment of this culture is largely the responsibility of a facilitator, who can moderate posts and ensure the tone of interactions is respectful and appropriate [22]. Thus, the role of a facilitator can be demanding because building trust and administering the network are as important as sharing knowledge. The high demands of the role were anticipated by two participants in this study who commented that the facilitator should either be “someone with plenty of time” or “the role should be rotated.” When implementing a GP training network, facilitation needs to account for the demands of administration, maintenance of a supportive culture, and provision of some topic expertise. It may also be desirable to share these roles among different facilitators.

The establishment of clear goals for a VCoP is seen as an important motivator for uptake [16]. In the HOBE study [17], primary care providers in Spain were invited to a VCoP for the Basque region with the agreed-upon goal of developing and implementing innovations in primary care. As a result, a range of innovations were developed and then implemented. In this study, some specific goals such as *helping registrars to pass exams* and *learning how to put guidelines into practice* were deemed important. However, factor analysis showed that this group of goals could be seen as a single factor, which was labeled *usefulness for training*. The generalized linear regression showed that this *usefulness* factor was the key independent predictor of intention to actively use the network. Thus, it appears that the network should be useful for training as its overall goal, rather than focusing only on, for example, passing exams. The review of the comments by users showed that this usefulness largely fell into two categories of training support: support for knowledge transfer and professional support to overcome isolation. These two concepts are likely linked because barriers to knowledge sharing, such as time, geography, and the structure of the workplace, can lead to professional isolation [3].

The importance of perceptions of usefulness as drivers of intention to use is consistent with the technology acceptance model, in which uptake of a technology is driven by its perceived usefulness, and usefulness as a driver is even stronger than ease of use [24]. Perceived usefulness was higher among GP registrars than supervisors, as was their intention to use a VCoP for training purposes. This finding is in keeping with a previous study in which intention to use a VCoP for GP training was shown to be independently linked to the training level of the registrar, with the most junior registrars indicating the highest intention to use the VCoP [18]. However, the finding contrasts in some ways with a US study in which social media usage by doctors was associated with being younger, male, and having teaching hospital privileges [25]. Although the study explored a different set of technology tools, the contrasting findings suggest there is more to learn about the factors affecting adoption of technology tools by doctors. Finally, the quality of the content was seen as an important driver for use of the VCoP. This suggests that the quality of the content may influence the perception of usefulness. Whether the relation between the uptake of social media and an intention to use technologies for training purposes can be explained by training stage, age, quality of content, or other variables requires further investigation. However, understanding what is perceived as useful for the target participants of a VCoP remains a key factor in VCoP design.

Therefore, in the establishment of a VCoP for GP training it will be important to focus on the usefulness for supervisors and registrars. Supervisors may need more convincing about the usefulness of the VCoP than registrars and in fact the VCoP may ultimately be more useful for registrars than supervisors. However, promoting the perception of usefulness to the potential participants may encourage uptake. The perceived usefulness will rest on clear goals of improved support for knowledge sharing and overcoming professional isolation. It may even be that supervisor perception of usefulness could increase if registrars use such a VCoP and find that it achieves these goals.

A broad church of users is acknowledged as an important factor for success from the literature [6,16] because a knowledge gradient is important to effective knowledge transfer. In this study, this breadth was supported by respondents, with both registrars and supervisors clearly favoring the inclusion of all levels of GP registrars and supervisors. However, the inclusion of specialists and allied health professionals was more favored by registrars than supervisors. This disparity may be a reflection of different expectations of supervisors and registrars. Registrars may feel that specialized providers will give them more knowledge; however, supervisors may feel that they, as senior GPs, are the best providers of the types of knowledge that a GP in training will need. This difference in expectation between registrars and supervisors was also evident in the different ranking of levels of support needed and the topics of need. Although there were significant differences between registrars and supervisors for some topics (eg, cardiology), the overall trend was for topics with a large *tacit* knowledge component to be ranked more highly. Topics such as managing a consultation, compensable injury medicine, and certifying someone as fit to drive all involve a high degree of *know-how* (ie, tacit knowledge), as well as *know-what* (ie, explicit knowledge). The transfer of tacit knowledge is seen as a particular strength of VCoPs, in which knowledge is not only imparted, but is discussed and subsequently implemented in a user’s practice, rather than simply being passed on [26]. When implementing a VCoP for GP training, important elements will include the exact breadth of the *church*, the alignment of knowledge needs and expectations among participants where possible, an acknowledgement of different needs for different groups where needs do not align, and a focus on the benefits of tacit knowledge transfer.

According to the health VCoP framework, another important aspect of a VCoP for health is *Step 7: Technology and Community*. From this study, the preference for a mix of face-to-face and online interactions was highlighted by the difference between building trust and meeting training needs. Most respondents preferred to build trust face-to-face, but the reverse was true for training, with respondents largely preferring online training. This is supported by the literature in which participants are more likely to build trust online through prior face-to-face contact [26]. Online environments are sometimes seen as more impersonal, as facial cues and body language can be missed, making it more difficult to build trust [27]. It has been suggested that online trust building may be improved by creating trust in the organization through integrity and openness [26]. There may also be a role for improving trust by transmitting body language and facial cues with emerging applications such as video chat. In contrast to this, training online is quite appropriate for information transfer, which can efficiently take place online when required. The need to build trust online perhaps also explains the preference for users to use their own names and have a private, password-protected site, rather than an open, pseudonym-based site. When implementing a VCoP for GP training, help and basic information may be provided online, but trust will ideally be built face-to-face, augmented potentially by video applications and the credibility of the network itself.

### Conclusions

The findings of the health framework for VCoPs are relevant when developing an implementation plan for a VCoP for GP training. The implementation plan should involve following the seven steps of facilitation: stakeholder engagement, developing clear goals, engaging a *broad church* of users, creating a supportive environment, using benchmarking and feedback, providing a range of online tools, and establishing online and face-to-face community engagement to transfer knowledge and build trust. Some additional considerations are that the facilitator role may be split between several members to provide administrative as well as expert support, training can be online but trust may be better off initially built face-to-face, and knowledge expectations and needs of supervisors and registrars need to be aligned where possible and addressed separately where needs differ. Most importantly, such a network needs to provide high-quality content and be perceived as useful to drive usage. All of these steps aim to drive uptake of the network and facilitate knowledge sharing, thus improving connectedness and overcoming professional isolation.

The sharing of knowledge to overcome professional isolation and improve connectedness is a useful goal for a VCoP. GP training can be isolating, leading to issues of workforce retention in rural areas. If professional isolation can be overcome, this may assist with the training and ultimately the retention of rural and regional general practitioners. This has broader implications beyond the training of rural general practitioners in Australia; this may inform training of medical specialists and allied health professionals as they rotate through regional placements, both in Australia and in other countries attempting to train and retain health professionals across a wide geography.

### Limitations

There are a number of limitations to this study. Firstly, the study was conducted in a single regional training provider. This may introduce bias around demographics and geography which could limit the generalizability of the findings. However, in terms of rural and urban comparisons, the study participants were evenly distributed across rural and urban areas, with no significant differences found based on rurality, so this may improve the confidence in the external validity of the studies.

Secondly, the response rate for the surveys was 40%, but the overall numbers were modest. Response rates to physician surveys are often lower than those for nonphysicians, but the response rate here is still a little lower than the 40-50% quoted in a review of physician response rates [15]. This lower response rate may mean that there is self-selection bias, with users more interested in this area more likely to response to a survey, and thus the generalizability of the results may be affected. Methods to improve response rates were used, including a personal message from the author and a nonmonetary incentive; however, the literature notes that monetary incentives and shorter questionnaires have higher response rates, and the questionnaires in this study were quite lengthy [15].

There may be a self-selection bias in this study, as it was a study regarding online attitudes and the survey was distributed via email with a survey link. This online distribution method may have encouraged responses from users with higher baseline levels of confidence with online communication. In spite of this potential bias, the overall levels of confidence and usage were at least in keeping with, if not below, the levels found in some comparative studies, such as a recent study on social media usage among physicians in the United States [16], indicating that any bias may not be large.

Finally, it is important to acknowledge the dynamic nature of technology. Since this survey was conducted in October 2011, new versions of technology tools have been developed with increasing functionality. The technology knowledge and skills of medical practitioners has also evolved during this period. The finding should, therefore, be read with this in mind. Despite this, the tools discussed in this study remain the foundation of many online interactions and the conceptual model we discuss can be applied to any set of technologies.

## Appendix

Multimedia Appendix 1 Study survey given to participants.

## Abbreviations

CCCGPT: Coast City Country General Practice Training
CoP: community of practice
GP: general practice
IT: information technology
VCoP: virtual community of practice
