# Supplementary material for: A Virtual Community of Practice for General Practice Training: A Preimplementation Survey
Source: JMIR Med Educ. 2016 Aug 18;2(2):e13. doi: 10.2196/mededu.5318 (PMC5041368; doi:10.2196/mededu.5318)
Supplement: Multimedia Appendix 1 [file mededu_v2i2e13_app1.pdf]

Participant Information Sheet

Investigating GP Registrar perceptions regarding the usefulness of medical online communities

CCCGPT ONLINE NETWORK PRIORITY AREAS

PARTICIPANT INFORMATION SHEET

Dr Stephen Barnett, Prof Sandra Jones, Prof Don Iverson, Mr Lance Barrie

The Centre for Health Initiatives at the University of Wollongong, NSW, invites you to take part in research looking at General Practitioner/Medical Educator and Registrar perceptions regarding the usefulness and design of an online medical community within CoastCityCountry Training (CCCT). This research is funded by CoastCityCountry General Practice Training (CCCGPT).

WHY IS THE RESEARCH IMPORTANT?

Online medical communities are becoming prominent and highly active overseas. http://www.sermo.com/ in the US has over 70 000 physicians using it. The Canadian Medical Association has launched http://www.asklepios.ca/ and in the UK there is http://www.doctors.net.uk/.Theses networks can be a very useful educational resource - http://www.sermo.com/ has had over 30 000 discussions listed, with over 2 million posts from users in the last 18 months. This network of users allows the sharing of clinical and non-clinical information between providers.

This survey builds on existing research by the researchers about GP and GPR experiences of online collaborative education using online communities, as well as expectations about its possible future usefulness. The aim of this survey is to refine the priorities identified in the previous phase of research regarding an online communication platform within CCCGPT. Furthermore, it is hoped that insight will be provided on the preferences of the online communication platform.

WHAT WILL BE ASKED OF ME IF I AGREE TO PARTICIPATE IN THE RESEARCH PROJECT?

You will be asked to complete a brief survey. In the survey, you will be asked to indicate your attitudes to a number of different aspects of an online platform within CCCT. The survey will take ten minutes to complete.

At the end of the survey, you have the option of entering the draw to win an iPad2. If you would like to enter the draw you are asked to leave contact details, however these details will remain confidential and will not be used for any other purpose.

WILL MY INFORMATION BE KEPT CONFIDENTIALLY?

The information you provide will be completely confidential, and individual data will not be identifiable in any publications. If you choose to enter the competition, all contact details will be removed from our database once a winner has been chosen.

Your participation is entirely voluntary. Your decision to participate or not participate will not affect your relationship with your medical practice or the University of Wollongong. By completing survey, your consent will be assumed. At the conclusion of the survey you will be asked to submit or withdraw your responses from the analysis stage.

WHAT WILL THE DATA BE USED FOR?

The results will be used to make evidence based recommendations to assist communication processes between GPs/Medical Educators and Registrars. Research findings will be presented in journal papers and presentations to allow dissemination of the findings.

In order to participate in this research you will need to click on the link in the email and complete the online survey.

For further information you can contact one of the researchers below at the Centre for Health Initiatives, The University of Wollongong, telephone (02) 4221 4456.

Mr Lance Barrie lanceb@uow.edu.au
Dr Stephen Barnett sbarnett@bsmp.com.au

If you have any questions about the conduct of this research, you may contact the University of Wollongong Human Research Ethics Committee on 02 4221 4457.

1. I have been provided with information about this project and agree to participate in this research. I understand that I can withdraw or discontinue at any time and that my participation is completely voluntary.

- ◯ Yes
- ◯ No

2. What is your sex?

3. What is your age? (in years)

4. Is the practice you are primarily in classified as a rural or general term?

5. How long have you been a part of the CCCT organisation? (in years)

6. I am a:

7. REGISTRARS need help in KNOWING the latest best practice guidelines in the following subject areas

	Strongly Disagree	Disagree	Neither disagree or agree	Agree	Strongly Agree
Hypertension	◯	◯	◯	◯	◯
Diabetes	◯	◯	◯	◯	◯
COPD	◯	◯	◯	◯	◯
Back pain	◯	◯	◯	◯	◯
Management of skin disorder	◯	◯	◯	◯	◯
Management of skin cancer	◯	◯	◯	◯	◯
Mental Health	◯	◯	◯	◯	◯
ECG interpretation	◯	◯	◯	◯	◯
Radiology	◯	◯	◯	◯	◯

Other (please specify)

8. REGISTRARS need help in IMPLEMENTING the latest best practice guidelines in these subject areas

	Strongly Disagree	Disagree	Neither disagree or agree	Agree	Strongly Agree
Hypertension	◯	◯	◯	◯	◯
Diabetes	◯	◯	◯	◯	◯
COPD	◯	◯	◯	◯	◯
Back pain	◯	◯	◯	◯	◯
Management of skin disorder	◯	◯	◯	◯	◯
Management of skin cancer	◯	◯	◯	◯	◯
Mental Health	◯	◯	◯	◯	◯
ECG interpretation	◯	◯	◯	◯	◯
Radiology	◯	◯	◯	◯	◯

Other (please specify)

9. Please indicate your degree of agreement with the following statements about your educational needs:
I need help in KNOWING the latest best practice guidelines in these subject areas

	Strongly Disagree	Disagree	Neither disagree or agree	Agree	Strongly Agree
Asthma	◯	◯	◯	◯	◯
Hypertension	◯	◯	◯	◯	◯
Diabetes	◯	◯	◯	◯	◯
COPD	◯	◯	◯	◯	◯
Back pain	◯	◯	◯	◯	◯
Management of skin disorders	◯	◯	◯	◯	◯
Management of skin cancer	◯	◯	◯	◯	◯
Mental Health	◯	◯	◯	◯	◯
ECG interpretation	◯	◯	◯	◯	◯
Radiology	◯	◯	◯	◯	◯

Other (please specify)

10. I need help in IMPLEMENTING the latest best practice guidelines in these subject areas

	Strongly Disagree	Disagree	Neither disagree or agree	Agree	Strongly Agree
Asthma	◯	◯	◯	◯	◯
Hypertension	◯	◯	◯	◯	◯
Diabetes	◯	◯	◯	◯	◯
COPD	◯	◯	◯	◯	◯
Back pain	◯	◯	◯	◯	◯
Management of skin disorder	◯	◯	◯	◯	◯
Management of skin cancer	◯	◯	◯	◯	◯
Mental Health	◯	◯	◯	◯	◯
ECG interpretation	◯	◯	◯	◯	◯
Radiology	◯	◯	◯	◯	◯

Other (please specify)

11. Please rate the following statements:

	Strongly Disagree	Disagree	Neither Agree or Disagree	Agree	Strongly Agree
I am comfortable sharing my knowledge with colleagues from CCCT	◯	◯	◯	◯	◯
I am comfortable sharing my knowledge with those outside CCCT	◯	◯	◯	◯	◯
I am confident that the knowledge I share is correct and accurate	◯	◯	◯	◯	◯
I make time to exchange knowledge with colleagues	◯	◯	◯	◯	◯
I would rather others gain their own knowledge	◯	◯	◯	◯	◯
I am confident that others will listen to my experiences	◯	◯	◯	◯	◯

12. Please indicate which of the following you would prefer.

◯ a. I would prefer to share knowledge with colleagues in CCCTGP purely online communication

◯ b. I would prefer to share knowledge with colleagues in CCCTGP with a mixture of face-to-face and online communication

◯ c. I would prefer to share knowledge with colleagues in CCCTGP purely face-to-face

13. Please indicate which of the following you would prefer.

◯ a. I would prefer professional support with colleagues in CCCTGP purely online communication

◯ b. I would prefer professional support with colleagues in CCCTGP with a mixture of face-to-face and online communication

◯ c. I would prefer professional support with colleagues in CCCTGP purely face-to-face

14. Please rate the following statements:

	Strongly Disagree	Disagree	Neither Agree or Disagree	Agree	Strongly Agree
I am comfortable sharing my knowledge with colleagues from CCCT	◯	◯	◯	◯	◯
I am comfortable sharing my knowledge with those outside CCCT	◯	◯	◯	◯	◯
I am confident that the knowledge I share is correct and accurate	◯	◯	◯	◯	◯
I make time to exchange knowledge with colleagues	◯	◯	◯	◯	◯
I would rather others gain their own knowledge	◯	◯	◯	◯	◯
I am confident that others will listen to my experiences	◯	◯	◯	◯	◯

15. Please indicate which of the following you would prefer.

◯ a. I would prefer to share knowledge with colleagues in CCCTGP purely online communication

◯ b. I would prefer to share knowledge with colleagues in CCCTGP with a mixture of face-to-face and online communication

◯ c. I would prefer to share knowledge with colleagues in CCCTGP purely face-to-face

16. Please indicate which of the following you would prefer.

◯ a. I would prefer professional support with colleagues in CCCTGP purely online communication

◯ b. I would prefer professional support with colleagues in CCCTGP with a mixture of face-to-face and online communication

◯ c. I would prefer professional support with colleagues in CCCTGP purely face-to-face

A Community of Practice is a group of doctors sharing knowledge and support. They can be facilitated by technology such as shared online discussions, resources and messaging.

17. If such an online network were available in Coast City Country, how important to YOU are the following practical outcomes from sharing knowledge with peers and experts using an online network?

	Not Important	Slightly Important	Somewhat Important	Important	Very Important
Assist registrars pass exams	◯	◯	◯	◯	◯
Help me put guidelines into practice through discussion with colleagues	◯	◯	◯	◯	◯
Help registrars put guidelines into practice through discussion with colleagues	◯	◯	◯	◯	◯
Provide me shared resources to support my teaching of registrars	◯	◯	◯	◯	◯
Provide me with colleague support and advice in managing registrars	◯	◯	◯	◯	◯
Help me keep up to date through discussion with peers	◯	◯	◯	◯	◯
Provide me with professional support	◯	◯	◯	◯	◯
Build better links with topic experts	◯	◯	◯	◯	◯

Is there anything else you would like to add

A Community of Practice is a group of doctors sharing knowledge and support. They can be facilitated by technology such as shared online discussions, resources and messageing.

18. If such an online network were available in Coast City Country, how important to YOU are the following practical outcomes from sharing knowledge with peers and experts using an online network?

	Not Important	Slightly Important	Somewhat Important	Important	Very Important
Help registrars Pass exams	◯	◯	◯	◯	◯
Feel more confident in my medical skills	◯	◯	◯	◯	◯
Learn from colleagues how to put guidelines into practice	◯	◯	◯	◯	◯
Feel more supported in general practice	◯	◯	◯	◯	◯
Develop a broader network of colleagues	◯	◯	◯	◯	◯

Other, please specify

19. How important is it that this community is formally sponsored and supported by CCCTGP?

- ◯ Not Important
- ◯ Slightly Important
- ◯ Somewhat Important
- ◯ Important
- ◯ Very Important

20. How would you best build trust in the identity and expertise of other users in a Virtual Community?

	Strongly Disagree	Disagree	Neither agree or disagree	Agree	Strongly Agree
Through prior knowledge of the member	◯	◯	◯	◯	◯
Online profile, qualifications and description of the member	◯	◯	◯	◯	◯
Face-to-face meetings/talks to online interaction	◯	◯	◯	◯	◯

21. If you would like face-to-face meetings, would they be:

- ◯ Part of usual meetings eg training days
- ◯ Specific Community 'events'
- ◯ I do not think face to face meetings are necessary

22. How likely is it that you will do the following:

	Not Likely	Slightly Likely	Somewhat Likely	Likely	Very Likely
Actively participate eg post new topics, upload resources, facilitate discussions	◯	◯	◯	◯	◯
Share knowledge eg answer questions or make comments on other doctors' topics	◯	◯	◯	◯	◯
Read but not contribute	◯	◯	◯	◯	◯
Not use the network	◯	◯	◯	◯	◯

23. What would most encourage you to keep participating?

	Strongly Disagree	Disagree	Neither agree or disagree	Agree	Strongly Agree
The value of the online content	◯	◯	◯	◯	◯
Strength of the relationships developed with other members	◯	◯	◯	◯	◯
Financial reward for participation	◯	◯	◯	◯	◯
Professional recognition by the network for participation (eg. An online points system with incentives such as gift vouchers)	◯	◯	◯	◯	◯
CPD points for participation	◯	◯	◯	◯	◯

Other (please specify)

24. Who would you like to have involved in the community from CCCT?

- [ ] Registrars
- [ ] Supervisors
- [ ] Educators
- [ ] Administration (non medical)
- [ ] Nil

Other (please specify)

25. Who would you like to have involved in the community from outside CCCT?

- [ ] non-CCCTGP registrars
- [ ] Medical Students
- [ ] Allied health
- [ ] University academics
- [ ] Practice Nurses
- [ ] Medical Specialists (If yes, then which specialties and, if specific names then please state name and specialty)

Other suggestions or specialty/name of specialist

26. How often would you like to see that another member has added information onto the site?

- ◯ Every day
- ◯ 3-4 times a week
- ◯ 1-2 a week
- ◯ Fortnightly
- ◯ Monthly

27. How often do you think you might interact with such a site?

- ◯ Every day
- ◯ 3-4 times a week
- ◯ 1-2 a week
- ◯ Fortnightly
- ◯ Monthly

28. Would you like to receive an email every time a topic that you have posted on receives comment or has been updated?

- ◯ Yes
- ◯ No
- ◯ Not sure

29. How important are the following items for the online platform?

Training needs for using the site:

	Not Important	Slightly Important	Somewhat Important	Important	Very Important
Online Help (text topics and images)	◯	◯	◯	◯	◯
Help Desk (telephone)	◯	◯	◯	◯	◯
Formal face-to-face training session	◯	◯	◯	◯	◯
Online tutorials	◯	◯	◯	◯	◯

30. How important are the following items for the online platform?

Collaborative ideas:

	Not Important	Slightly Important	Somewhat Important	Important	Very Important
Discussion Forum (all users)	◯	◯	◯	◯	◯
Discussion Forum (Private for specific users only)	◯	◯	◯	◯	◯
Live Chat	◯	◯	◯	◯	◯
Shared Documents/guidelines etc	◯	◯	◯	◯	◯
Videoconferencing	◯	◯	◯	◯	◯
Email List	◯	◯	◯	◯	◯

Other (please specify)

31. In an online platform, what is your preference?

- ◯ Use a pseudonym
- ◯ Use your real name
- ◯ Choice of either

32. Which of the following would you prefer the online platform to be?

- ◯ Secure password protected
- ◯ Public and no password

33. How important is it that the community has formal leadership/facilitation?

○ Not important ○ Slightly Important ○ Somewhat Important ○ Important ○ Very Important

34. Who would be an ideal person to lead and monitor the online network?

- ☐ Topic expert
- ☐ GPR liaison officer
- ☐ GP supervisor
- ☐ CCCT admin
- ☐ Online network developer

Other (please specify)

35. If you would like to go in the draw to win an iPad 2, please leave your contact details below:

Name

Email

Contact Phone Number
